# Case report: Patiromer-induced hypercalcemia 

**DOI:** 10.5414/CNCS109782

**Published:** 2019-08-09

**Authors:** Michael R. Wiederkehr, Ankit N. Mehta, Michael Emmett

**Affiliations:** Texas A&M Health Science Center, College of Medicine, Baylor University Medical Center at Dallas, Division of Nephrology, Department of Internal Medicine, Dallas, TX, USA

**Keywords:** patiromer, hypercalcemia, hyperkalemia, potassium binding agents

## Abstract

Patiromer is a novel potassium-binding compound which has recently received FDA approval. This ion exchange resin releases calcium when it binds potassium. We describe the development of hypercalcemia after initiation of patiromer. The calcium levels fell when the drug was stopped but recurred when it was later resumed. Patiromer was again discontinued, and the serum calcium level fell back into the normal range. We believe this patient manifested patiromer-induced hypercalcemia.

## Introduction 

Hyperkalemia is an increasingly frequent clinical problem. This is largely due to the growing use of medications that blunt renal potassium excretion. They include angiotensin-converting enzyme inhibitors, angiotensin receptor blockers, and mineralocorticoid receptor antagonists. Hyperkalemia is more likely to complicate the use of these medications in patients with a reduced glomerular filtration rate (GFR). However, these drugs have become standard of care for many patients with chronic kidney and chronic heart disease. Consequently, interventions to treat and prevent hyperkalemia are becoming more important. 

Until recently, the only available gastrointestinal (GI) potassium-binding drug was sodium polystyrene sulfonate, a drug with questionable efficacy and significant safety concerns [1]. The FDA approved patiromer calcium, an organic, non-absorbable, insoluble cation exchange polymer in 2015. Patiromer binds potassium, and to a lesser extent, hydrogen and other cations, while releasing calcium. In 2018, the FDA approved sodium zirconium cyclosilicate (ZS-9), an insoluble, tasteless, and odorless inorganic crystalline salt that binds potassium and smaller amounts of other cations such as ammonium, calcium, and magnesium, in exchange for the release of sodium (and much less hydrogen). The calcium released from patiromer and the sodium released from ZS-9 is absorbed to varying degrees by the GI tract, and the potential adverse consequences of these systemic loads have been studied and generally deemed acceptable [1, 2] 

Most of the absorbed calcium load from patiromer is excreted into the urine. This may raise the risk of calcium-containing kidney stones [3, 4]. However, with a reduced GFR, the increment in urinary calcium excretion is blunted and the theoretical possibilities of hypercalcemia and ectopic calcifications have been raised [1, 5]. Two cases of hypercalcemia developing after patiromer initiation have been reported in abstract form [6, 7]. 

## Case presentation 

A 48-year-old Caucasian woman had developed Henoch-Schönlein purpura during childhood. This resulted in stable chronic kidney disease (CKD) with creatinine levels fluctuating between 2.2 and 2.5 mg/dL, normal serum electrolytes, and low-grade proteinuria (< 1 g/day). She took lisinopril/hydrochlorothiazide 20/25 mg with excellent blood pressure control. 

Proteinuria increased to 2.7 g/day, and spironolactone 25 mg daily was initiated in an effort to reduce proteinuria and stabilize renal function [8]. Because the potassium level was in the high-normal range, she was instructed to follow a low potassium diet, and patiromer 8.4 g daily was initiated. At that time, her serum calcium was 9.4 mg/dL. We planned to follow chemistries carefully, but she missed several follow-up appointments. She returned after 4 months complaining of generalized weakness. Potassium was normal, but creatinine had increased to 2.7 mg/dL, and calcium was 11.4 mg/dL. Vitamin D studies were normal, and she was not taking any calcium supplements. She was instructed to follow a low calcium diet. Hydrochlorothiazide was changed to low-dose torsemide, but 1 month later, calcium had increased further to 12.8 mg/dL and creatinine to 4.2 mg/dL (Table 1). 

Patiromer, lisinopril, torsemide, and spironolactone were all discontinued, and over the next 2 weeks, calcium fell to 9.8 mg/dL and creatinine to 2.8 mg/dL. Realizing her risk for progression of CKD, we resumed lisinopril with patiromer, but calcium again increased from 9.8 to 10.5 mEq/L. Lisinopril and patiromer were stopped, and her calcium returned to 9.8 mg/dL (Table 1). 

## Discussion 

Hypercalcemia likely developed as a result of systemic absorption of calcium released from patiromer. Although the risk of hypercalcemia was raised as a potential complication of patiromer [5], it was not seen in any of the published clinical trials of this drug [9, 10, 11]. The reported adverse effects included flatulence, diarrhea, worsening of hypertension or CKD, hypoglycemia, hypokalemia, and hypomagnesemia. 

Emmett et al. [4] recently discussed the three most likely pathways for the calcium released from patiromer: (a) GI absorption with subsequent excretion into the urine or systemic retention, (b) binding to anions such as oxalate and phosphate within the GI lumen with fecal excretion, and (c) rebinding to the resin itself. Bushinsky et al. [3] demonstrated that patiromer produces a dose-dependent decrease in urinary excretion of potassium, magnesium, and sodium with a concomitant dose-dependent increase in urinary calcium. 

In the late 1960s, multiple studies reported the development of hypercalcemia when patients with renal insufficiency were treated with various calcium-charged potassium-binding resins [12, 13, 14, 15, 16], and this complication continues to be reported [17]. 

The package insert for patiromer (Veltassa; Relypsa, Inc., Redwood City, CA, USA) instructs clinicians to monitor serum potassium and magnesium levels but does not mention serum calcium [18]. 

Although the development of hypercalcemia in patients using patiromer must be uncommon, it is not surprising that it may occur in some patients. We speculate that these patients have some underlying pathophysiology or a clinical disorder that makes them susceptible to this development. Patients with more advanced CKD, and thus less robust calciuria, are at higher risk. It is also likely that other disorders associated with hypercalcemia, such as hyperparathyroidism, sarcoidosis, or certain malignancies, would increase the risk of this complication. We recommend that serum calcium levels be carefully monitored in patients receiving chronic patiromer therapy. 

## Funding 

None. 

## Conflict of interest 

The authors declare no financial conflict of interest with any products from Relypsa, Inc., the manufacturer of patiromer, have never received honoraria from the company, and own no stock in the company. 

**Table 1.** Table 1.

Time points	A	B	C	D	E	F	G	H	I
Date	12/18/17	12/29/18	05/07/18	6/6/2018	06/12/18	06/20/18	07/26/18	08/03/18	09/12/18
Patiromer		---------------------------------------------------------→				---------------------→			
Spironolactone		---------------------------------------------------------→							
Lisinopril/HCTZ	------------------------------------------------------------------------→								
Lisinopril/torsemide				-------------------→					
Lisinopril						---------------------→			
Protein, total, 24 h Ur		2,724 mg/24 h							
Protein/creat ratio	2,815 mg/g crea		2,225 mg/g crea			760 mL/g crea			
Creatinine, serum	2.26 mg/dL	2.01 mg/dL	2.73 mg/dL	4.23 mg/dL	3.92 mg/dL	2.82 mg/dL	5.47 mg/dL	4.07 mg/dL	3.4 mg/dL
Potassium, serum	5.3 mmol/L	4.8 mmol/L	4.7 mmol/L	4.4 mmol/L	5.1 mmol/L	4.8 mmol/L	5.5 mmol/L	4.4 mmol/L	4.8 mmol/L
Calcium, serum	10.1 mg/dL	9.4 mmg/dL	11.4 mg/dL	12.8 mmol/L	11.1 mg/dL	9.8 mg/dL	10.5 mg/dL	9.8 mg/dL	10.0 mg/dL
Phosphorus, serum	3.0 mg/dL	3.2 mg/dL	4.4 mg/dL	6.1 mg/dL	6 mg/dL	3.6 mg/dL	5.6 mg/dL	5.0 mg/dL	3.7 mg/dL
Albumin, serum	4.1 g/dL	3.7 g/dL	4.2 g/dL	4.9 g/dL	4.3 g/dL	4 g/dL	4.4 g/dL	4.0 g/dL	4.2 g/dL
PTH, intact	48 pg/mL		26 pg/mL	39 pg/mL					
Vitamin D, 25-Hydroxy				51.2 ng/mL					
Calcitriol (1,25 [OH]2 D)				16.0 pg/mL					
